# Robotic surgery for colorectal cancer

**DOI:** 10.1002/ags3.12007

**Published:** 2017-04-25

**Authors:** Chu Matsuda, Yosuke Adachi

**Affiliations:** ^1^ Department of Gastroenterological Surgery Graduate School of Medicine Osaka University Suita Japan; ^2^ Center for the Study of Medical Education School of Medicine Kurume University Kurume Japan

Robotic surgery (RS) for colorectal disease was first reported in 2002.^1^ Since then, many studies of RS have been widely reported. Technical advantages of the da Vinci robotic system could overcome the limitation of laparoscopic surgery (LS) for colorectal cancer, by giving the surgeon a 3D view, no tremor, better ergonomics, enhanced dexterity, precision and a short learning curve.

Recently, several meta‐analyses of randomized controlled trials to compare the safety and efficacy of RS and LS have been reported. Trastulli *et al*. identified eight non‐randomized studies that included a total of 858 patients who underwent surgery for rectal cancer with 344 (40.2%) in the RS group and 510 (59.7%) in the LS group. Meta‐analysis suggested that the conversion rate to open surgery with RS was significantly lower than that with LS (odds ratio [OR] = 0.26, 95% confidence interval [CI]: 0.12–0.57, *P* = 0.0007). There were no significant differences in operation time, length of hospital stay, time to resume regular diet, postoperative morbidity and mortality, and oncological accuracy of resection.^2^

Lin *et al*. also conducted a meta‐analysis and reported that RS had favorable outcomes considering conversion compared with LS for rectal cancer. Meanwhile, the following factors were similar between RS and LS: operation time, blood loss, days to passing flatus, length of hospital stay, complications and pathological details, including number of lymph nodes harvested, distal resection margin, and positive circumferential resection margin.^3^

Liao *et al*. identified four randomized controlled studies for meta‐analysis. In total, 110 patients underwent colorectal surgery in the RS group and 116 in the LS group. The results revealed that blood loss, conversion rate and time to recovery of bowel function in the RS group were significantly lower than those in the LS group. There were no significant differences in complication rates, length of hospital stay, proximal margins, distal margins and harvested lymph nodes between the two techniques.^4^

Based on the review of these meta‐analyses, RS for colorectal cancer has a lower conversion rate compared with LS, with no difference in recovery, postoperative and oncological outcomes. In contrast, there are few reports on long‐term prognosis of RS for colorectal cancer, although recent survival analysis using propensity score matching shows that the 5‐year survival rates of robotic versus laparoscopic resection were 91% versus 78% for overall survival and 73% versus 68% for disease‐free survival.^5^

The ROLARR trial^6^ is a pan‐world, prospective, randomized, controlled, unblinded, superiority trial enrolling over 400 patients to compare RS versus standard LS for the curative treatment of rectal cancer. The results presented at the European Society of Coloproctology in 2015 showed that there was no difference in terms of oncological clearance, perioperative morbidity, mortality and conversion to open surgery between the two groups. However the trial is yet to be published, and the results of long‐term outcomes are yet to be analyzed. This trial will provide further information about the efficacy of RS compared with LS and may give us the final decision for RS of rectal cancer.

RS for colorectal cancer is still under development and will improve instrumentation and haptic feedback with advances in technology. The most important point is whether or not RS is superior to LS in oncological outcome and patients’ quality of life. The high cost of RS is another problem. If these problems are clarified, the status of RS in rectal cancer will be promising.
